# Successful Hepatectomy for a Liver Abscess With Portal Vein Thrombus and Hepatic Artery Dissection: A Case Report

**DOI:** 10.7759/cureus.78367

**Published:** 2025-02-01

**Authors:** Norifumi Iseda, Tomohiro Iguchi, Seiya Kato, Noriaki Sadanaga, Hiroshi Matsuura

**Affiliations:** 1 Department of Surgery, Saiseikai Fukuoka General Hospital, Fukuoka, JPN; 2 Department of Pathology, Saiseikai Fukuoka General Hospital, Fukuoka, JPN

**Keywords:** common bile duct stones, hepatic artery dissection, hepatic resection, liver abscess, portal vein thrombus

## Abstract

Liver abscesses can be associated with biliary disease and are occasionally accompanied by portal vein thrombosis. Hepatic artery obstruction has been reported to result from aneurysms, thrombosis, iatrogenic factors, and arterial dissection; however, to the best of our knowledge, no cases of liver abscess with obstruction of the portal vein and hepatic artery have been reported. A 51-year-old man presented with a chief complaint of heartburn. A detailed investigation revealed common bile duct stones and an 8-cm multilocular abscess in the left lobe of the liver. Choledocholithiasis was achieved by endoscopic retrograde cholangiopancreatography. Despite antibiotic treatment with meropenem, his symptoms and inflammatory reaction did not improve, and computed tomography (CT) revealed obstruction of the left branch of the portal vein and left hepatic artery. Because it proved difficult to control his infection, we performed a left hepatic lobectomy. Histopathological examination of the operative specimen revealed a hepatic abscess with portal vein thrombosis and left hepatic artery dissection. The postoperative course was favorable, and the patient was discharged on postoperative day eight. Thirty months later, he continues to do well. We here report a patient with a liver abscess with portal vein thrombus and hepatic artery dissection whose infection was successfully controlled by hepatectomy.

## Introduction

Liver abscesses can be defined as pus-filled cavities surrounded by a fibrous capsule by contamination with and multiplication of microorganisms within healthy or diseased liver parenchyma [1]. The incidence of liver abscesses ranges from 2.30 to 17.59 per 100,000 individuals annually and is increasing worldwide [2-3]. Age, antibiotic use, comorbidities such as diabetes, underlying hepatobiliary diseases such as cholangitis and cirrhosis, and regular use of proton pump inhibitors may be associated with the development of liver abscesses [4]. Biliary disease is also one of the most common causes of liver abscesses worldwide [5]. Several cases of liver abscesses with portal vein thrombosis have been reported. Hepatic artery obstruction has been reported to result from aneurysms, thrombosis, iatrogenic factors, and arterial dissection [6,7]; however, to the best of our knowledge, no cases of liver abscess with obstruction of the portal vein and hepatic artery have been reported. We here present a patient with a liver abscess with portal vein thrombus and hepatic artery dissection who successfully underwent left hepatic lobectomy.

## Case presentation

A 51-year-old man was admitted to Saiseikai Fukuoka General Hospital in Fukuoka, Japan, for fever, heartburn, and fatigue during the previous week. He had no notable medical history. On admission to our hospital, his body temperature was 38°C, blood pressure was 119/72 mmHg, and pulse rate was 127 beats per minute. The abdomen was flat and soft with no tenderness. Laboratory findings were as follows: white blood cell count, 24,200/µL; C-reactive protein, 29.48 mg/dL; platelet count, 249×103/µL; total bilirubin, 15 mg/dL; direct bilirubin, 12 mg/dL; pancreatic amylase, 57 U/L; prothrombin time, 70%; and activated partial thromboplastin time, 42.9 sec (Table 1).

**Table 1:** Laboratory investigations CBC: complete blood count; WBC: white blood cells; RBC: red blood cells; ALT: alanine transaminase; AST: aspartate transaminase; ALP: alkaline phosphatase; AMY: amylase; CRP: C-reactive protein; PT: prothrombin time; INR: international normalized ratio; APTT: activated partial thromboplastin time

Test	Observed value	Reference range
CBC		
Hemoglobin	13.5 g/dL	13.7-16.8 g/dL
WBC	24.2 x 10^3^/µL	3.3-8.6 x 10^3^/µL
Platelets	249 x 10^3^/µL	158-348 x 10^3^/µL
RBC	4.6 x 10^6^/µL	4.3-5.5 x 10^6^/µL
Biochemical test		
Total bilirubin	15 mg/dL	0.4-1.5 mg/dL
Direct bilirubin	12 mg/dL	0.0-0.4 mg/dL
ALT	266 U/L	10-42 U/L
AST	95 U/L	13-30 U/L
ALP	713 U/L	106-322 U/L
AMY	57 U/L	44-132 U/L
Total protein	5.7 g/dL	6.6-8.1 g/dL
Albumin	1.9 g/dL	4.1-5.1 g/dL
Blood urea nitrogen	14.9 mg/dL	8-20 mg/dL
Serum creatinine	0.8 mg/dL	0.6-1.0 mg/dL
CRP	29.5 mg/dL	0.0-0.1 mg/dL
Coagulation profile		
PT	11 %	80-120 %
INR	1.1	0.8-1.1
APTT	42 seconds	24-39 seconds

**Figure 1:**
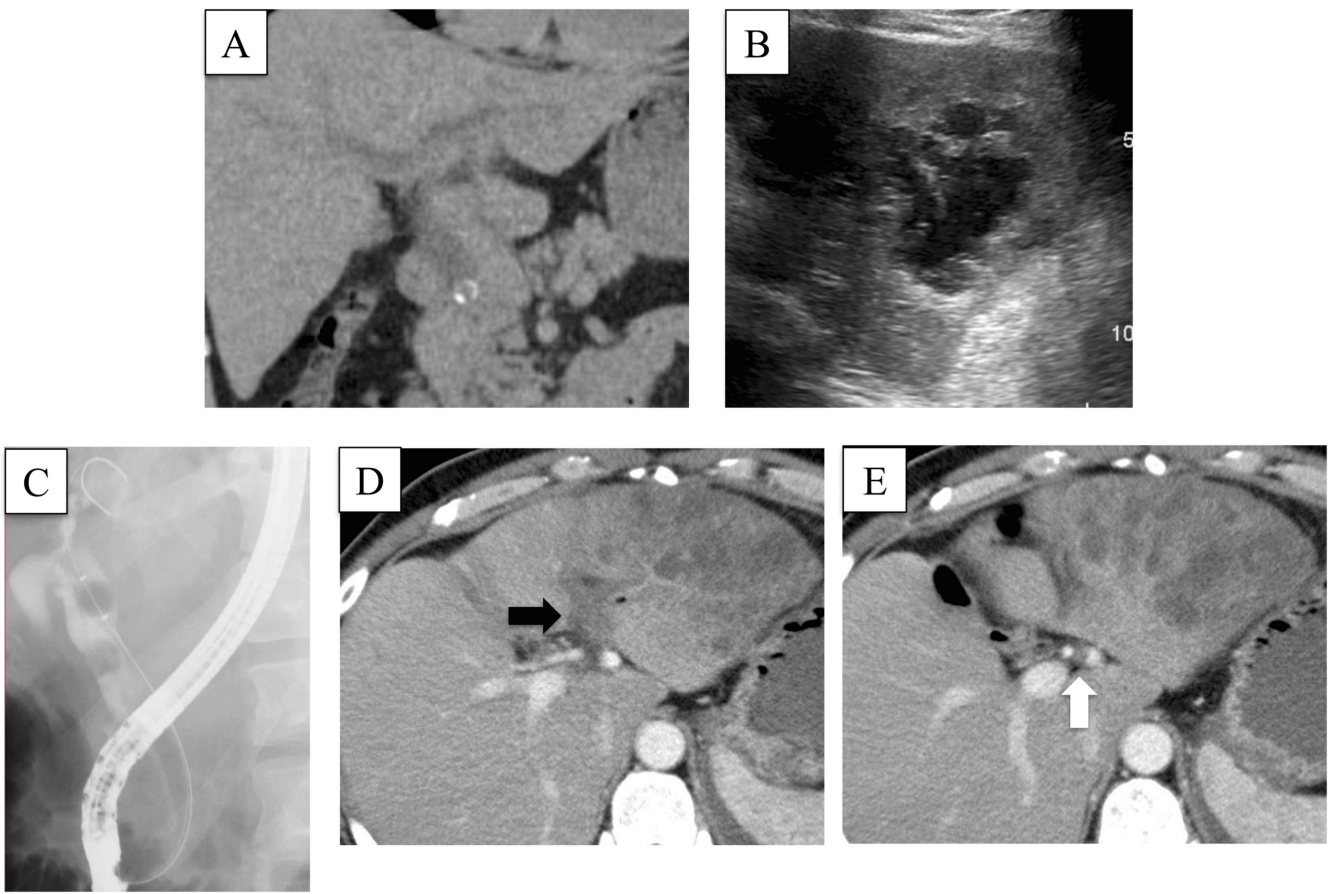
Inspection results (A) Computed tomography image revealing dilated bile ducts proximal to obstructing common bile duct stones; (B) Ultrasound image showing a multilocular, low-echoic, 8-cm lesion diagnosed as a left hepatic lobe abscess; (C) Endoscopic retrograde cholangiopancreatography image showing stones in the common bile duct with dilatation; (D and E) Contrast-enhanced CT scan images revealing that the left branch of the portal vein (black arrow) and the left hepatic artery (white arrow) are obstructed proximal to the infarction.

Computed tomography (CT) (Figure 1A) and ultrasound examination (Figure 1B) revealed stones in the retropancreatic common bile duct and a multilocular low-echoic 8-cm-diameter lesion in the left hepatic lobe. We diagnosed a left hepatic lobe abscess associated with choledocholithiasis and performed endoscopic retrograde cholangiopancreatography. Radiographs confirmed multiple choledocholithiasis with a dilated common bile duct. We proceeded with sphincterotomy, which resulted in the spontaneous propulsion of some stones, the remainder being extracted with a balloon extractor (Figure 1C). Bile cultures yielded *Klebsiella pneumoniae*. Treatment with intravenous meropenem resulted in gradual improvement in his inflammatory response, but his malaise and anorexia continued. Repeat CT with contrast enhancement showed obstruction of the left branch of the portal vein (Figure 1D) and left hepatic artery (Figure 1E), liver infarction, and a liver abscess (Figure 1D).

**Figure 2:**
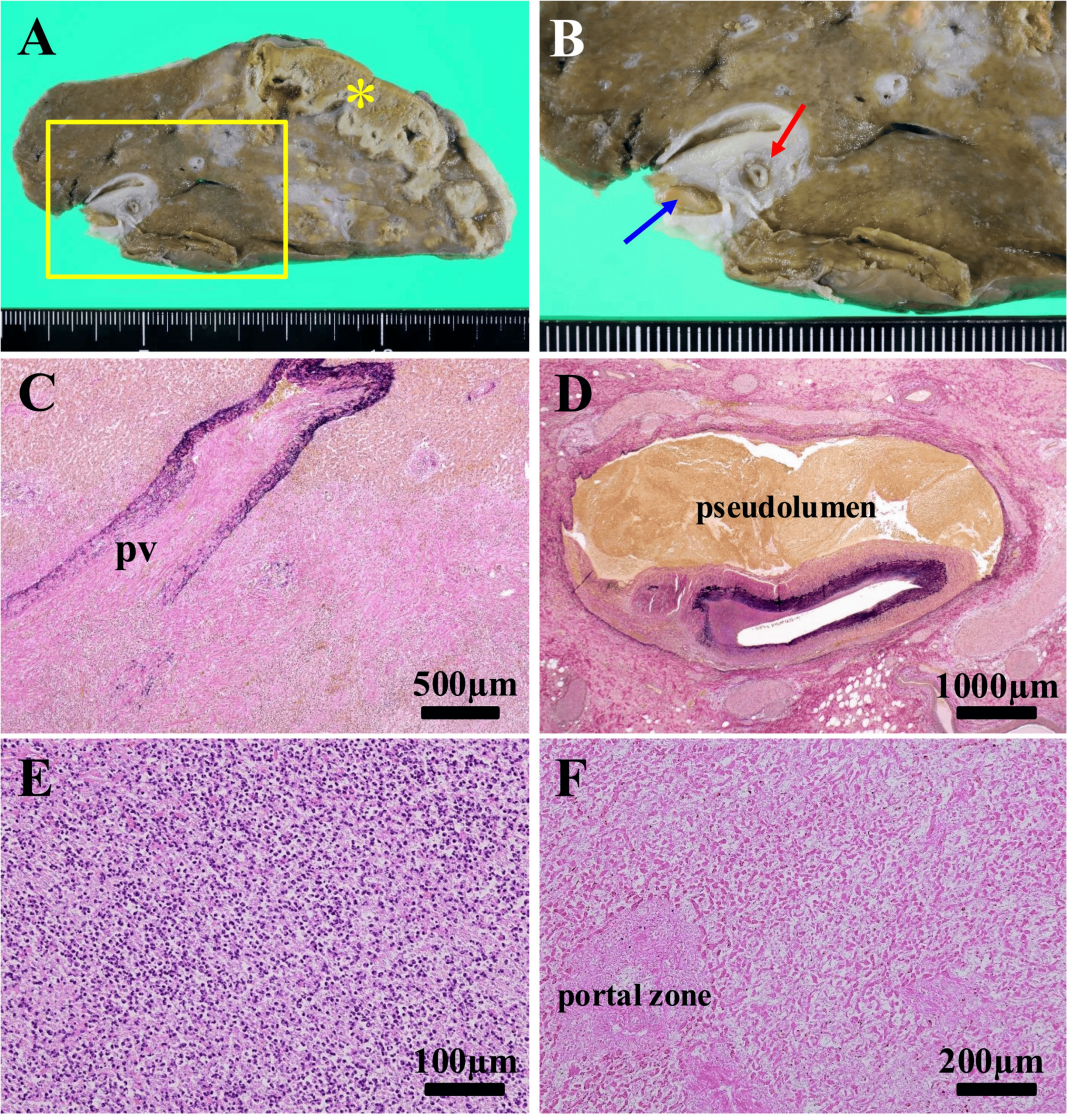
Macroscopic and microscopic findings (A) Gross appearance of the cut surface. An abscess and area of infarction are located peripherally (*). (B) Enlarged view of the area enclosed by the rectangle in A showing a portal vein thrombosis (blue arrow) and dissection of the hepatic artery (red arrow); (C) Photomicrograph of the portal vein obstruction with evidence of inflammation (Elastica van Gieson stain); (D) Photomicrograph of hepatic artery dissection (Elastica van Gieson stain). The pseudolumen is labeled. (E) Photomicrograph of the liver abscess showing abundant neutrophilic infiltration and liquefaction (hematoxylin and eosin stain). (F) Photomicrograph of the area of liver infarction. Coagulation necrosis of liver parenchyma is evident (hematoxylin and eosin stain).

We assumed that the thrombus had been caused by severe inflammation and was responsible for the obstructions of the left branch of the portal vein and left hepatic artery. Because retrograde infection after papillary incision can be difficult to eradicate, we considered that surgery was required to control the infection. We therefore performed a left hepatic lobectomy. The operation time was 247 minutes, and the blood loss was 1,037 mL. The liver was blackish with a slight yellow discoloration and a smooth surface, and there was an abscess in the lateral segment. There were strong adhesions at the origin of the left hepatic artery. We accordingly severed it distal to its origin at the site of obstruction. We dissected the portal vein with the Glissonean sheath because it was difficult to separate its left branch from the surrounding tissue. The postoperative course was uneventful, and the patient was discharged on postoperative day eight. Pathological examination of the operative specimen revealed a necrotic focus and infarction with an indistinct border in the peripheral liver parenchyma and accompanying cholestasis and irregular fibrosis (Figure 2A). Additionally, portal vein thrombosis (Figure 2B) and dissection of the hepatic artery were noted (Figure 2B). Histological examination showed thrombotic obstruction of the portal vein with extensive suppurative inflammation (Figure 2C) and hepatic artery dissection with compression by the pseudolumen (Figure 2D). Consistent with the macroscopic findings, histological examination revealed a liver abscess with abundant neutrophilic infiltration and liquefaction (Figure 2E) and coagulation necrosis of infarcted liver parenchyma (Figure 2F). As a result of the success of this treatment, the patient has a good quality of life, having remained free of major problems for 30 months since the surgery.

## Discussion

Liver abscesses leading to portal vein thrombus have been reported and are considered rare [8]. The mechanism of thrombus formation associated with biliary tract infection is as follows: (i) circulating cytokines cause vascular endothelial damage; (ii) further endothelial damage in adjacent blood vessels is caused by acute cholecystitis or cholangitis; and (iii) hypercoagulation associated with bacterial infection develops [9]. In the present case, thrombus formation in the portal vein may have been associated with direct spread of infection from the biliary system to adjacent blood vessels (Figure 2C) or circulating cytokines rather than with a systemic disorder.

Regarding the obstruction of the left hepatic artery, preoperative CT showed no evidence of dissection or aneurysm, suggesting that the obstruction was thrombotic. Histopathological examination of the operative specimen revealed arterial dissection and arterial obstruction having been caused by pressure from the pseudolumen (Figures 2B, 2D). Dissection of the hepatic artery is so rare that its incidence has not yet been clearly established [10]. Weakness of the tunica media leads to arterial dissection, the weakness of this part of the arterial wall most likely being attributable to structural abnormalities of elastic fibers, which are the main component of the tunica media [11]. A new form of cell death, ferroptosis, has recently attracted attention. Ferroptosis is characterized by iron-dependent accumulation of lethal lipid reactive oxygen species [12, 13]. Chen et al. have shown that perivascular inflammation causes degeneration of arterial tunica media by ferroptosis, which is caused by iron-dependent accumulation of lethal lipid reactive oxygen species, leading to progressive arterial dissection [14]. Arterial dissection can block the point of branching off of another artery, blocking blood flow [15]. In the present case, preoperative CT failed to identify arterial dissection, possibly indicating that it occurred subsequently. Alternatively, our difficulty in assessing the arterial obstruction may have hindered the identification of dissection. Although the possibility that the surgical manipulation exogenously caused the arterial dissection cannot be denied, we assumed that inflammation had been spread to the left hepatic artery, which may have led to the weakening of the arterial wall and dissection. The combination of occlusion of the hepatic artery and of the portal vein system would have blocked the feeding vessels to the injured liver tissue, likely resulting in infarction and a refractory liver abscess. To our knowledge, this is the first report of a liver abscess with both portal vein thrombus and hepatic artery dissection.

Traditionally, treatment of liver abscesses consists of antibiotic administration, drainage of purulent collections, and hepatectomy. Methods of drainage include an open surgical approach, laparoscopic drainage, radiographically guided percutaneous drainage, percutaneous aspiration, and needle aspiration [16,17]. The current first-line treatment for liver abscesses is sonographic- or CT-guided needle puncture and aspiration. Needle aspiration enables identification of the causative microorganism and may incidentally reveal evidence of biliary tract communication. After the insertion of a drain, the collection can be completely evacuated (5). Surgical drainage of liver abscesses has a role in cases of failed percutaneous treatment, large abscesses (>5 cm), and/or multilocular abscesses [18, 19]. Hepatectomy can be considered for liver abscess in selected cases, such as when the abscess is not accessible for CT-guided percutaneous drainage or has ruptured, or the patient has failed to improve with optimal medical therapy and percutaneous drainage [20]. In the present case, percutaneous puncture was not performed before surgery because the liver abscess was huge, measuring 8 cm, and multilocular. A further justification for choosing to perform a hepatectomy was that infection would have been difficult to control in the presence of liver ischemia.

## Conclusions

We report a rare case of liver abscess with portal vein thrombus and hepatic artery dissection treated by surgical resection. The combination of occlusion of the hepatic artery and of the portal vein system would have blocked the feeding vessels to the injured liver tissue, likely resulting in infarction and a refractory liver abscess. Surgical drainage of liver abscesses has a role in cases of failed percutaneous treatment, large abscesses (>5 cm), and/or multilocular abscesses. Surgical treatment can be successful in patients with an intractable liver abscess and such rare vascular abnormalities. In cases of refractory liver abscess, it is important to confirm portal and arterial blood flow, as both may be blocked.

## References

[1] Chiche L, Dargère S, Le Pennec V, Dufay C, Alkofer B (2008). Pyogenic-liver abscess: diagnosis and management (Article in French). Gastroenterol Clin Biol.

[2] Pfister D, Núñez NG, Pinyol R (2021). NASH limits anti-tumour surveillance in immunotherapy-treated HCC. Nature.

[3] Meddings L, Myers RP, Hubbard J (2010). A population-based study of pyogenic liver abscesses in the United States: incidence, mortality, and temporal trends. Am J Gastroenterol.

[4] Yin D, Ji C, Zhang S (2021). Clinical characteristics and management of 1572 patients with pyogenic liver abscess: a 12-year retrospective study. Liver Int.

[5] Lardière-Deguelte S, Ragot E, Amroun K (2015). Hepatic abscess: diagnosis and management. J Visc Surg.

[6] Halasz NA (1991). Cholecystectomy and hepatic artery injuries. Arch Surg.

[7] Tanabe G, Kawaida K, Hamanoue M (1999). Treatment for accidental occlusion of the hepatic artery after hepatic resection: report of two cases. Surg Today.

[8] Valla DC, Condat B (2000). Portal vein thrombosis in adults: pathophysiology, pathogenesis and management. J Hepatol.

[9] DeLeve LD, Valla DC, Garcia-Tsao G (2009). Vascular disorders of the liver. Hepatology.

[10] Crowhurst TD, Ho P (2011). Hepatic artery dissection in a 65-year-old woman with acute pancreatitis. Ann Vasc Surg.

[11] Nakashima Y (2010). Pathogenesis of aortic dissection: elastic fiber abnormalities and aortic medial weakness. Ann Vasc Dis.

[12] Dixon SJ, Lemberg KM, Lamprecht MR (2012). Ferroptosis: an iron-dependent form of nonapoptotic cell death. Cell.

[13] Iseda N, Itoh S, Toshida K (2022). Ferroptosis is induced by lenvatinib through fibroblast growth factor receptor-4 inhibition in hepatocellular carcinoma. Cancer Sci.

[14] Chen Y, Yi X, Wei X, Jiang DS (2022). Ferroptosis: a novel pathological mechanism of aortic dissection. Pharmacol Res.

[15] Nienaber CA, Eagle KA (2003). Aortic dissection: new frontiers in diagnosis and management: Part II: therapeutic management and follow-up. Circulation.

[16] Johannsen EC, Sifri CD, Madoff LC (2000). Pyogenic liver abscesses. Infect Dis Clin North Am.

[17] Israeli R, Jule JE, Hom J (2009). Pediatric pyogenic liver abscess. Pediatr Emerg Care.

[18] Hope WW, Vrochides DV, Newcomb WL, Mayo-Smith WW, Iannitti DA (2008). Optimal treatment of hepatic abscess. Am Surg.

[19] Tan YM, Chung AY, Chow PK, Cheow PC, Wong WK, Ooi LL, Soo KC (2005). An appraisal of surgical and percutaneous drainage for pyogenic liver abscesses larger than 5 cm. Ann Surg.

[20] Boustany A, Obed A, AbuAssi M, Bashir A, Jarrad A (2022). Role of hepatectomy in patients with liver abscess: a single center experience. Am J Gastroenterol.

